# Appraisal of guidelines and variations in recommendations on drug therapy for invasive aspergillosis prevention and treatment

**DOI:** 10.3389/fphar.2025.1443487

**Published:** 2025-03-31

**Authors:** Jing Wang, Ju Sun, Da-Wei Yang, Hai-Shan Wang, Wei Meng, Hong-Yan Li

**Affiliations:** ^1^ Department of Pharmacy, Qindao University Medical College Affiliated Yantai Yuhuangding Hospital, Yantai, China; ^2^ Department of Liver Transplantation, The Affiliated Hospital of Qindao University, Qindao, China; ^3^ P.E. Teaching and Research Group, Yantai No.1 Middle School of Shandong, Yantai, China; ^4^ Department of Intensive Care Unit, Yantai YEDA Hospital, Yantai, China; ^5^ Department of Cardiology, Qindao University Medical College Affiliated Yantai Yuhuangding Hospital, Yantai, China

**Keywords:** invasive aspergillosis, therapeutics, antifungal agents, practice guideline, AGREE II

## Abstract

**Background:**

In recent years, many guidelines related to aspergillosis have been published worldwide. However, no studies have applied assessment tools to systematically evaluate the quality of these guidelines.

**Objectives:**

This study aimed to assess the quality of clinical practice guidelines and compared their recommendations related to drug therapy for the prevention and treatment of invasive aspergillosis.

**Methods:**

Electronic databases, guideline development organizations, and professional society websites were searched to identify clinical practice guidelines for invasive aspergillosis published between 1 January 2013, and 12 September 2023. The Appraisal of Guidelines Research and Evaluation (AGREE) II instrument was used to evaluate the quality of the guidelines. Recommendations for the drug prevention and treatment of invasive aspergillosis were extracted and descriptively analyzed.

**Results:**

Among the 18 included clinical practice guidelines, the median scores and interquartile range for each AGREE II domain were: scope and purpose, 76.39% (69.1%, 80.21%); stakeholder involvement, 59.72% (50.35%, 67.02%); rigor of development, 64.58% (44.4%, 72.27%); clarity and presentation, 81.25% (68.06%, 91.32%); applicability, 41.67% (36.46%, 47.92%); and editorial independence, 76.05% (50%, 87.5%). Voriconazole and isavuconazole are recommended as first-line therapy for invasive aspergillosis currently. Posaconazole remains the first choice for invasive aspergillosis prophylaxis in patients with hematological malignancies.

**Conclusion:**

The development processes and reporting of invasive aspergillosis -related clinical practice guidelines varied and their quality requires improvement. The guideline recommendations have changed since the approval of isavuconazole.

## 1 Background

Invasive aspergillosis (IA) is an important cause of morbidity and mortality in immunocompromised patients. IA is common in patients with acute leukemia (AL), allogeneic hematopoietic stem cell transplantation (HSCT) (Girmenia et al., 2014) and solid organ transplantation (SOT) (Singh and Husain, 2013) and less common in patients with chronic obstructive pulmonary disease (COPD), chronic granulomatous disease (King et al., 2016), medical intensive care, and severe burns. Invasive pulmonary aspergillosis (IPA) also occurs secondary to coronavirus disease-19 (COVID-19) (Alanio et al., 2020). The Global Action for Fungal Infections (Global Action for Fungal Infections, 2023) reports that >30 million people are at risk for corticosteroids or other therapies, and >300,000 patients develop IA annually. The management of aspergillosis is a critical and challenging medical issue.

Clinical practice guidelines (CPGs) are the essence of evidence-based medicine, and the quality of their development determines their benefits for clinical practice and patients. Many guidelines related to aspergillosis have been published worldwide in recent years; however, no studies have applied assessment tools to systematically evaluate the quality of these guidelines. The Appraisal of Guidelines for REsearch and Evaluation (AGREE) II instrument is used to assess the methodological rigor and transparency with which a guideline is developed (Brouwers et al., 2010).

We comprehensively searched for IA-related guidelines published in the past decade, evaluated their quality using the AGREE II instrument, and compared their recommendations for IA treatment and prevention. As IPA is the most frequent manifestation of IA, the treatment of other forms of IA is usually based on IPA drug therapy in combination with surgical resection of necrotic lesions; therefore, we extracted key recommendations for IPA or IA treatment and prevention.

## 2 Methods

### 2.1 Search strategy

We searched the PubMed, Embase, Cochrane Library, Web of Science, and three major Chinese (Wanfang Data, SinoMed and China National Knowledge Infrastructure Database) academic databases from 1 January 2013, to 12 September 2023, without language restrictions. We also searched the National Institute for Health and Clinical Excellence (NICE), Guidelines International Network (GIN), World Health Organization (WHO), Scottish Intercollegiate Guidelines Network (SIGN), and National Health and Medical Research Council (NHMRC) websites. The three main search terms were “invasive fungal disease (IFD),” “IPA,” and “CPG.” Keywords and medical subject headings were searched, with the search results for IFD and IPA first combined using the “OR” operator, followed by the application of the “AND” operator with the results for GCP. The search strategy in PubMed is detailed in the supplementary material. Additionally, we reviewed the references and websites in the guidelines.

### 2.2 Guideline identification

The guideline inclusion criteria were: (1) evidence-based guidelines reporting on search strategies, literature quality or data extraction, and classification of the level of evidence (LOE) and strength of recommendation (SOR); (2) guidelines containing clear recommendations for the prevention or treatment of IPA or IA in a prominent section as an independent disease rather than mixed with other IFD; and (3) for guidelines issued by the same organization, only the updated version was included.

The exclusion criteria were: (1) guidelines for chronic pulmonary aspergillosis, allergic bronchopulmonary aspergillosis, extrapulmonary aspergillosis, and aspergillosis in children; (2) guidelines relating only to the diagnosis of aspergillosis, environmental surveillance, patient management, antifungal stewardship, and the utility of certain antifungal drugs; and (3) guideline interpretations, review articles, conference summaries, consensus statements without recommendations, and secondary publications (including versions translated from other languages).

### 2.3 Data collection

Two reviewers independently screened the studies according to the inclusion and exclusion criteria. Any disagreements were resolved through discussions. Relevant data were extracted using previously developed forms, including the general characteristics and grading systems of the CPGs. Recommendations related to IA prophylaxis or treatment were also extracted for comparison.

### 2.4 Quality assessment

Four assessors independently assessed the CPGs using the AGREE II instrument. The AGREE II consists of 23 key items organized into six quality domains. Each domain captures a unique dimension of guideline quality; namely,: scope and purpose, stakeholder involvement, rigor of development, clarity of presentation, applicability, and editorial independence. Each of the AGREE II items is rated on a seven-point scale (1-strongly disagree, 7-strongly agree). The quality score is calculated for each of six domains. Domain scores are calculated by summing the scores of the individual items in a domain and scaling the total as a percentage of the maximum possible score for that domain $\left(\frac{\left(\text{obtained}\;\text{score}-\text{minimum}\;\text{possible}\;\text{score}\right)}{\left(\text{maximum}\;\text{possible}\;\text{score}-\text{minimum}\;\text{possible}\;\text{score}\right)}\times 100\%\right)$ . Higher domain scores indicate better methodology and greater completeness of reporting in the corresponding CPG domain.

### 2.5 Statistical analysis

We used Microsoft Office 2022 (Microsoft Corp., Redmond, Washington, United States) to extract and analyze CPG information. Quantitative data statistics were obtained using IBM SPSS Statistics for Windows, version 26.0 (IBM Corp., Armonk, NY, United States). The intraclass correlation coefficient (ICC) with 95% confidence interval (CI) was used to evaluate the consistency among the assessors, with values <0.4 and >0.75 indicating poor and excellent consistency, respectively.

## 3 Results

### 3.1 Search results

**FIGURE 1:**
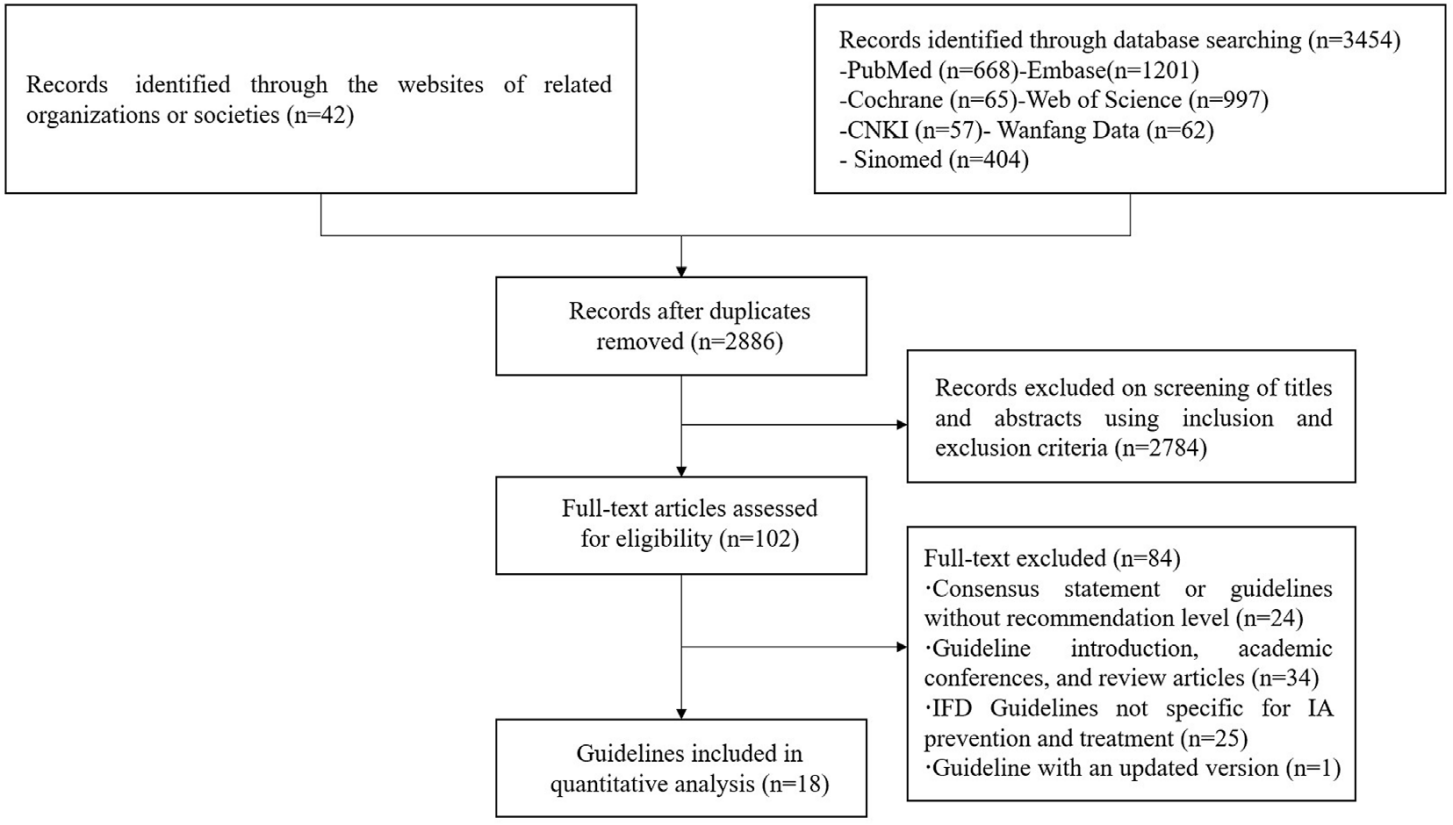
Flow chart of the identification process of CPGs for IPA.

Among 2857 relevant documents, 102 were eligible for full-text review, and 18 were included in the analysis. Figure 1 shows the flowchart of the guideline identification.

### 3.2 Characteristics of the included CPGs

All 18 included CPGs were evidence-based and classified according to the LOE and SOR. Eight CPGs were comprehensive IA guidelines without clearly defined target populations, five specialized in oncology or hematological patients, three in solid organ transplant recipients (SOTR), and two in patients with COVID-19-associated pulmonary aspergillosis (CAPA). Among recommendation themes, nine CPGs were related to IA, nine were IFD guidelines containing recommendations for aspergillosis. Twelve CPGs used the Grade of Recommendations, Assessment, Development, and Evaluation (GRADE) or modified GRADE system for evidence-level classification and recommendation synthesis, while six CPGs used self-defined or other grading systems. The general characteristics are presented in Table 1, and the grading systems of the guidelines are shown in the Supplementary Material.

**TABLE 1:** General characteristics of the included guidelines.

Guideline	Country	Developing organization	Target population	Theme of recommendations	Version
Taiwan, 2023 (Wu et al., 2023)	Taiwan	GREAT working group	COVID-19 patients	management and treatment of CABI, CAPA, CAC and CAM	first
Columbia, 2022 (Oñate et al., 2022)	Columbia	ACIN mycosis group	various populations	prophylaxis, treatment and prevention of IA	first
Australia, 2021 (Douglas et al., 2021)	Australia and New Zealand	—	haematology-oncology	diagnosis and management of IA	fourth
Global, 2021 (Verweij et al., 2021)	international	—	COVID-19 patients	epidemiology, diagnosis and management of CAPA	first
ASTCT, 2021 (Dadwal et al., 2021)	America	ASTCT/TID-SIG	HCTR	epidemiology, diagnosis, prophylaxis, and treatment of IA	second
Poland, 2020 (Gil et al., 2020)	Poland	PSHBT/PSPOH/PALSG	HM or HCTR	diagnosis, treatment, and prophylaxis of IFD	updated
AGIHO/DGHO, 2019 (Ruhnke et al., 2020)	Germany	AGIHO/DGHO	HM and/or ST	treatment of IFD	second
AST-IDCOP, 2019 (Husain and Camargo, 2019)	America	AST-IDCOP	SOTR	epidemiology, diagnosis, and management of Aspergillus	fourth
GEMICOMED-SEIMC, 2018 (Garcia-Vidal et al., 2019)	Spain	GEMICOMED/SEIMC	various populations	diagnostic, treatment, and prophylaxis of IA	updated
ESCMID-ECMM-ERS, 2017 (Ullmann et al., 2018)	Europe	ESCMID-ECMM-ERS	various populations	diagnosis, prophylaxis, treatment of IA	first
SWAB, 2017 (Kullberg et al., 2017)	Netherlands	SWAB	various populations	prophylaxis and treatment of IFD	second
ECIL-6, 2017 (Tissot et al., 2017)	Europe	ECIL	HM and HCTR	treatment of IFD	updated
IDSA, 2016 (Patterson et al., 2016)	America	IDSA	various populations	diagnosis, treatment, and prevention of IA	updated
Taiwan, 2016 (Kung et al., 2018)	Taiwan	IDST	various populations	treatment of IFD	third
China, 2016 (Shi BY, 2016)	China	OTBCMA/OTPBCMA	SOTR	diagnosis, prevention and treatment of IFD	second
Japan, 2014 (Kohno et al., 2016)	Japan	—	various populations	management of deep-seated mycosis	third
Middle East, 2014 (Al-Abdely et al., 2014)	Middle East	—	various populations	diagnosis, treatment, prophylaxis of IA	first
ESGICH, 2014 (Gavaldà et al., 2014)	Europe	ESGICH	SOTR	diagnosis, prevention and treatment of IFD	—

Abbreviation: CABI: COVID-19, associated bacterial infections; CAPA: COVID-19, associated pulmonary aspergillosis; CAC: COVID-19, associated candidiasis; CAM: COVID-19, associated mucormycosis; IA: invasive aspergillosis; HCTR: hematopoietic cell transplantation recipients; SOTR: solid organ transplant recipients; HM: hematological malignancies; ST: solid tumors; IFD: invasive fungal diseases; GREAT: Guidelines Recommendations for Evidence-based Antimicrobial agents use in Taiwan; ACIN: the Colombian Association of Infectious Diseases; ASTCT: american society for transplantation and cellular therapy; TID-SIG: transplant infectious disease special interest group; PSHBT: Polish Society of Hematology and Blood Transfusion; PSPOH: polish society of pediatric oncology and hematology; PALSG: polish adult leukemia study group; AGIHO/DGHO: the Infectious Diseases Working Party of the German Society of Hematology and Oncology; AST-IDCOP: american society of transplantation infectious diseases community of practice; GEMICOMED/SEIMC: the Study Group of Fungal Infections from the Spanish Society of Infectious Diseases and Clinical Microbiology; ESCMID: european society for clinical microbiology and infectious diseases; ECMM: the European Confederation of Medical Mycology; ERS: the European Respiratory Society; SWAB: stichting werkgroep antibioticabeleid; IDSA: the Infectious Diseases Society of America; IDST: the Infectious Diseases Society of Taiwan; OTBCMA: organ transplantation branch of the chinese medical association; OTPBCMA: organ transplantation physicians branch of the chinese medical association; ECIL: the European Conference on Infections in Leukemia; ESGICH: the ESCMID, study group for infections in compromised hosts.

### 3.3 Quality assessment

**FIGURE 2:**
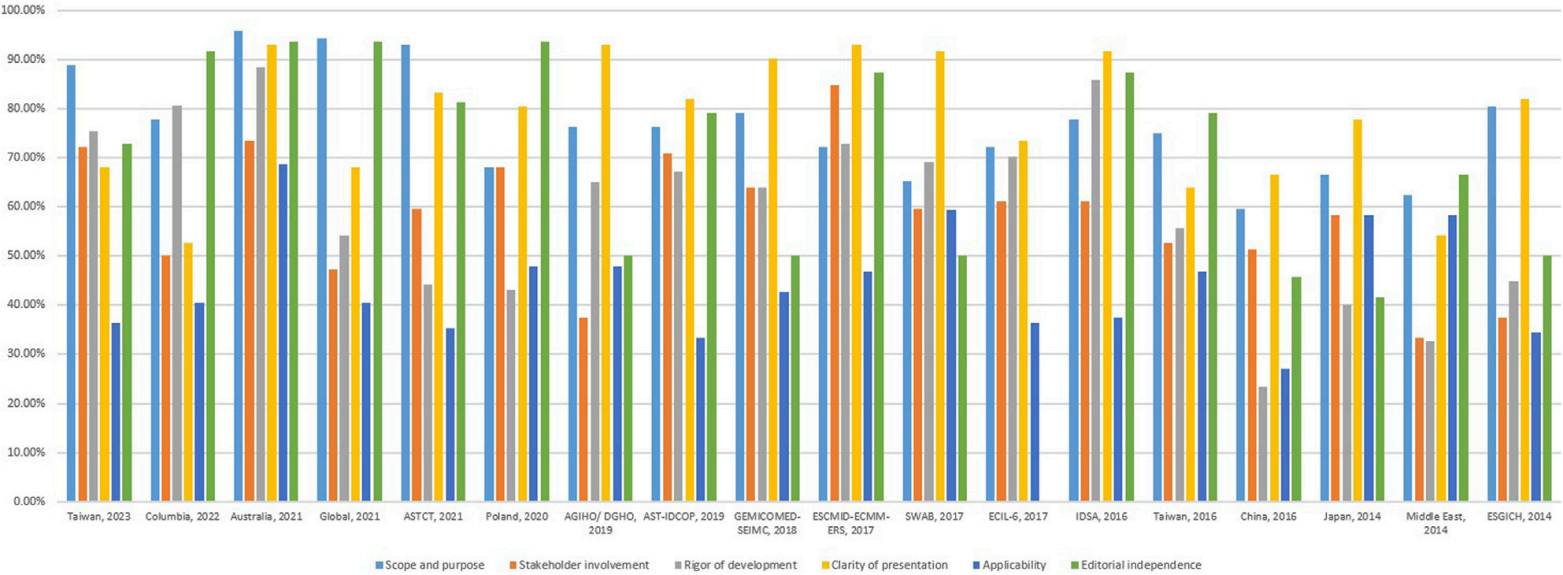
AGREE II Domain scores for included guidelines.

Four assessors independently evaluated the 18 CPGs. The ICC value was 0.83 (95%CI = 0.806–0.853), indicating high consistency among the assessors. Through the evaluation of the AGREE II assessment tool, significant differences were found in the quality of guidelines issued by different institutions. The mean domain score of the Australia 2021 guideline (Douglas et al., 2021) was the highest, while the lowest was in the China 2016 guideline (Shi BY, 2016). The median and interquartile range (IQR) of the six domains suggested relatively high scores for the clarity of presentation, scope and purpose, and editorial independence domains, while the applicability domain had the lowest score. The AGREE II domain scores of the included CPGs are presented in Table 2 and Figure 2.

**TABLE 2:** AGREE II Domain scores for included guidelines.

Guideline	Domain score (%)						Mean score (%)
	Scope and purpose	Stakeholder involvement	Rigor of development	Clarity of presentation	Applicability	Editorial independence	
Taiwan, 2023	88.89	72.22	75.52	68.06	36.46	72.92	69.01
Columbia, 2022	77.78	50	80.73	52.78	40.63	91.67	65.6
Australia, 2021	95.83	73.61	88.54	93.06	68.75	93.75	85.59
Global, 2021	94.44	47.22	54.17	68.06	40.63	93.75	66.38
ASTCT, 2021	93.06	59.72	44.27	83.33	35.42	81.25	66.17
Poland, 2020	68.06	68.06	43.23	80.56	47.92	93.75	66.93
AGIHO/DGHO, 2019	76.39	37.5	65.1	93.06	47.92	50	61.66
AST-IDCOP, 2019	76.39	70.83	67.19	81.94	33.33	79.17	68.14
GEMICOMED-SEIMC, 2018	79.17	63.89	64.06	90.28	42.71	50	65.02
ESCMID-ECMM-ERS, 2017	72.22	84.72	72.92	93.06	46.88	87.5	76.22
SWAB, 2017	65.28	59.72	69.27	91.67	59.38	50	65.89
ECIL-6, 2017	72.22	61.11	70.31	73.61	36.46	0	52.29
IDSA, 2016	77.78	61.11	85.94	91.67	37.5	87.5	73.58
Taiwan, 2016	75	52.78	55.73	63.89	46.88	79.17	62.24
China, 2016	59.72	51.39	23.44	66.67	27.08	45.83	45.69
Japan, 2014	66.67	58.33	40.1	77.78	58.33	41.67	57.14
Middle East, 2014	62.5	33.33	32.81	54.17	58.33	66.67	51.3
ESGICH, 2014	80.56	37.5	44.79	81.94	34.38	50	54.86
Median score	76.39	59.72	64.58	81.25	41.67	76.05	65.75
Interquartile range (IQR)	(69.1, 80.21)	(50.35, 67.02)	(44.4, 72.27)	(68.06, 91.32)	(36.46, 47.92)	(50, 87.5)	(58.27, 67.84)

#### 3.3.1 Scope and purpose

The median score (IQR) for this domain was 76.39% [69.1%, 80.21%; range 95.83% (Douglas et al., 2021)–59.72% (Shi BY, 2016)]. Almost all guidelines specifically described their overall objectives, with seven guidelines describing health problems phrased as questions (Patterson et al., 2016; Garcia-Vidal et al., 2019; Dadwal et al., 2021; Douglas et al., 2021; Verweij et al., 2021; Oñate et al., 2022; Wu et al., 2023).

#### 3.3.2 Stakeholder involvement

The median score (IQR) of this domain was 59.72% [48.61%, 65.98%; range 84.72% (Ullmann et al., 2018)–33.33% (Al-Abdely et al., 2014)]. Only Taiwan 2023 (Wu et al., 2023) included a methodologist in the professional groups. The guidelines barely mentioned the views and preferences of the target populations. Twelve CPGs clearly defined the target users.

#### 3.3.3 Developmental rigor

The median score (IQR) for this domain was 64.58% [44.4%, 72.27%; range 88.54% (Douglas et al., 2021)–23.44% (Shi BY, 2016)]. Ten CPGs scored >60% and clearly described the methods of forming recommendations, and there were explicit links between the recommendations and evidence. Six CPGs were externally reviewed by experts before their publication and six CPGs provided guideline update procedures, five of which mentioned both (Patterson et al., 2016; Ullmann et al., 2018; Husain and Camargo, 2019; Douglas et al., 2021; Oñate et al., 2022).

#### 3.3.4 Clarity of presentation

The median score (IQR) for this domain was 81.25% [68.06%, 91.32%; range 93.06% (Ullmann et al., 2018; Ruhnke et al., 2020; Douglas et al., 2021)–52.78% (Oñate et al., 2022)]. Only two CPGs scored <60% (Al-Abdely et al., 2014; Oñate et al., 2022); most guidelines clearly stated the recommendations and the key recommendations were easily identifiable.

#### 3.3.5 Applicability

The median score (IQR) for this domain was 41.67% (36.46%, 47.92%; range 68.75% (Douglas et al., 2021)–27.08% (Shi BY, 2016)). Most guidelines did not describe facilitators, barriers, or tools to promote their application; only two CPGs mentioned dissemination tools or teams to promote guideline implementation (Kullberg et al., 2017; Douglas et al., 2021).

#### 3.3.6 Editorial independence

The median score (IQR) of this domain was 76.05% [50%, 87.5%; range 93.75% (Gil et al., 2020; Douglas et al., 2021; Verweij et al., 2021)–0% (Tissot et al., 2017)]. Seven CPGs had no funding body statements, and one CPG did not report the competing interests of the guideline development group members (Tissot et al., 2017).

### 3.4 Recommendations for IA/IPA treatment

We extracted 515 recommendations related to IA/IPA treatment from the 18 included CPGs. The first options of primary therapy and combination therapy are listed in Table 3. The detailed treatment recommendations for each guideline can be found in the supplementary material.

**TABLE 3:** Treatment and prevention recommendations for IA or IPA.

Guideline	Primary therapy (most recommended)	Combination therapy	Prophylaxis for HM	Prophylaxis for SOTR
Taiwan, 2023	VCZ, ISZ, PCZ, L-AmB	VCZ + ANFG or CPFG, ISZ + L-AmB	—	—
Columbia, 2022	VCZ, ISZ	VCZ + an echinocandin	PCZ, VCZ	PCZ, VCZ, nebulized L-AmB
Australia, 2021	VCZ, ISZ, PCZ	VCZ + ANFG	—	—
Global, 2021	VCZ, ISZ, PCZ	-	—	—
ASTCT, 2021	VCZ, ISZ, L-AmB	an echinocandin + a triazole or L-AmB	PCZ, VCZ	—
Poland, 2020	VCZ, L-AmB, ISZ	L-AmB + VCZ or CPFG	PCZ	—
AGIHO/DGHO, 2019	VCZ, ISZ	VCZ + ANFG	—	—
AST-IDCOP, 2019	VCZ	—	—	echinocandins, VCZ, ITZ, nebulized AmB
GEMICOMED-SEIMC, 2018	VCZ, ISZ	VCZ + ANFG	PCZ, VCZ	echinocandins, ITZ, nebulized AmB
ESCMID-ECMM-ERS, 2017	ISZ, VCZ	VCZ + ANFG	PCZ	ITZ, nebulized AmB, echinocandins
SWAB, 2017	VCZ, ISZ	—	PCZ	—
ECIL-6, 2017	VCZ, ISZ	VCZ + ANFG	—	—
IDSA, 2016	VCZ	VCZ + an echinocandin	PCZ	VCZ, ITZ, nebulized AmB
Taiwan, 2016	VCZ	—	—	—
China, 2016	VCZ, L-AmB	VCZ + CPFG	—	—
Japan, 2014	VCZ	—	VCZ, MCFG	VCZ, L-AmB, echinocandins
Middle East, 2014	VCZ	—	PCZ	—
ESGICH, 2014	VCZ, L-AmB	VCZ + CPFG or ANFG	—	—

Abbreviation: VCZ: voriconazole; ISZ: isavuconazole; PCZ: posaconazole; L-AmB: liposomal Amphotericin B; ANFG: anidulafungin; CPFG: caspofungin; ITZ: itraconazole.

#### 3.4.1 Primary therapy

All guidelines recommend voriconazole with the highest level of evidence and recommendations for the primary treatment of IA/IPA. Guidelines published after 2016 recommended isavuconazole with an identical level of evidence as voriconazole or as an alternative to voriconazole for IA treatment. These guidelines recommended liposomal amphotericin B (L-AmB) as a first-line or alternative treatment. Six CPGs opposed the use of amphotericin B deoxycholate (D-AmB) due to the risk of adverse reactions. Eleven CPGs mentioned echinocandins alone or in combination as alternative primary therapies at a lower recommendation level, while two CPGs (Patterson et al., 2016; Garcia-Vidal et al., 2019) opposed their use. Nine CPGs recommended posaconazole as an alternative or second-line treatment for primary therapy, while one CPG (Garcia-Vidal et al., 2019) did not advocate its use. Amphotericin B lipid complex (ABLC), amphotericin B colloidal dispersion (ABCD), and itraconazole were weakly or not recommended by eight, five, and seven CPGs, respectively.

#### 3.4.2 Combination therapy

Twelve CPGs referred to combination therapy for IA/IPA. Four CPGs did not recommend routine primary combination therapy. Combination therapy was suggested for critically ill (Ruhnke et al., 2020; Douglas et al., 2021; Oñate et al., 2022) and high-risk (Patterson et al., 2016; Garcia-Vidal et al., 2019; Oñate et al., 2022) patients, in patients with suspected azole resistance (Douglas et al., 2021; Wu et al., 2023) or disseminated disease (Shi BY, 2016; Garcia-Vidal et al., 2019; Husain and Camargo, 2019; Oñate et al., 2022), or for salvage treatment of refractory IA (Garcia-Vidal et al., 2019; Oñate et al., 2022). The combination of triazole and echinocandin was the most commonly recommended.

#### 3.4.3 Salvage therapy

Ten CPGs described salvage treatments for refractory IA. The recommended principles included switching antifungal class (Gavaldà et al., 2014; Ullmann et al., 2018; Ruhnke et al., 2020; Douglas et al., 2021; Oñate et al., 2022), adding another antifungal drug to primary therapy (Patterson et al., Shi BY, 2016; Oñate et al., 2022), combination therapy (Gavaldà et al., 2014; Patterson et al., 2016; Ullmann et al., 2018; Douglas et al., 2021), and surgical management (Douglas et al., 2021; Oñate et al., 2022). SWAB 2017 (Kullberg et al., 2017) considered it to be of primary importance that azole resistance and a co-infection with Mucorales be excluded, on failure of voriconazole or isavuconazole. Australia 2021 (Douglas et al., 2021) stated that adequate triazole drug levels should be ensured. Other recommended measures included surgical resection of necrotic lesions (Douglas et al., 2021; Oñate et al., 2022), decrease or reversal of underlying immunosuppression (Oñate et al., 2022), use of an antifungal drug with an adverse effect profile that does not overlap with other co-administered drugs (Oñate et al., 2022). The medications recommended for salvage treatment include triazoles, echinocandins, and lipid formulations of amphotericin B (LFAB).

#### 3.4.4 Breakthrough infection

Six CPGs mentioned breakthrough infection of IA that occurred during fungal prevention. Switching to another class of antifungal agents (Patterson et al., 2016; Kung et al., 2018; Garcia-Vidal et al., 2019) and performing therapeutic drug monitoring (TDM) of triazoles (Patterson et al., 2016; Kung et al., 2018; Garcia-Vidal et al., 2019; Douglas et al., 2021) were the most recommended for breakthrough infections. Three CPGs (Patterson et al., 2016; Kung et al., 2018; Douglas et al., 2021) suggested antifungal susceptibility testing of Aspergillus isolates, two CPGs (Patterson et al., 2016; Kung et al., 2018) recommended reduction of underlying immunosuppression if feasible. Taiwan 2016 (Kung et al., 2018) also recommended reviewing potential interacting drugs, surgical resection of necrotic tissue and diagnostic approach for potential new etiology. Australia 2021 (Douglas et al., 2021) considered that if breakthrough IA occurred on triazole prophylaxis or therapy, a switch to L-AmB, and if on L-AmB therapy, a switch to voriconazole or isavuconazole was recommended (AⅢ).

#### 3.4.5 Treatment duration

Thirteen CPGs described the treatment duration for IA/IPA. Eight CPGs recommended a minimum treatment duration of 6–12 weeks, four CPGs recommended a course of at least 12 weeks. Consideration of the clinical and laboratory evidence of treatment response, site of infection, and degree and duration of immunosuppression were recommended to determine the course of treatment by eight, four, and nine CPGs, respectively.

### 3.5 Recommendations for IA/IPA prophylaxis

We extracted 200 recommendations related to IA prevention from 11 CPGs. The main preventive drugs are listed in Table 3. The detailed prevention recommendations of guidelines can be found in the supplementary material.

#### 3.5.1 IA prophylaxis for patients with prolonged neutropenia, HM, and/or HSCT

Nine CPGs described IA prevention strategies for high-risk patients with prolonged neutropenia, HM, and/or HSCT. As shown in Table 3, eight CPGs, except for Japan 2014 (Kohno et al., 2016), recommended posaconazole as the highest recommended level for primary prophylaxis in these patients, and all nine CPGs recommended voriconazole as an alternative drug at the same or lower recommended level. Other available drugs included itraconazole (Patterson et al., 2016; Garcia-Vidal et al., 2019; Oñate et al., 2022), echinocandins, and L-AmB (Ullmann et al., 2018; Garcia-Vidal et al., 2019; Oñate et al., 2022) at lower recommended levels.

#### 3.5.2 IA prophylaxis for SOT recipients

Six CPGs mentioned IA prevention in patients with SOT. Four CPGs (Ullmann et al., 2018; Garcia-Vidal et al., 2019; Husain and Camargo, 2019; Oñate et al., 2022) provided detailed descriptions of the conditions for IA prophylaxis in SOT recipients. The high-risk factors included *Aspergillus* colonization, graft rejection, augmented immunosuppression, reoperation, anastomotic problems, cytomegalovirus infection, renal replacement therapy, and hypogammaglobulinemia. The recommended regimens for IA prevention in patients undergoing lung transplantation include nebulized D-AmB, LFAB, voriconazole, itraconazole, posaconazole, isavuconazole, and echinocandins. IA prophylaxis could be performed in patients with other types of SOT after individualized risk assessment. The available drugs mainly included azoles, echinocandins, and L-AmB.

#### 3.5.3 Secondary prophylaxis

Eight CPGs referred to secondary prophylaxis for IA. All guidelines recommend the initiation of secondary prophylaxis in patients with previous IA requiring subsequent immunosuppression or during episodes of prolonged neutropenia.

## 4 Discussion

### 4.1 Requirements for improving guideline quality

Our systematic search and screening of guidelines for IA prevention and treatment published over the past decade analyzed 18 evidence-based CPGs. The quality of these guidelines varied. Although several guidelines described the application of AGREE II in the development process, some items did not score high after evaluation.

The problems in the scope and purpose domains included a lack of description of the health questions covered and the target populations. In the editorial independence domain, many guidelines failed to mention the funding body, and some did not state the role of the sponsors in developing the recommendations. In the stakeholder involvement domain, most guidelines focused on the inclusion of clinical experts in the guideline development group, with limited details on their personal information. Only one guideline mentioned methodological experts. Few guidelines considered the views and preferences of the target population during the development process. Although two guidelines mentioned this consideration, they did not provide detailed methods. Stakeholder participation can be ensured at different stages of guideline development through interviews or consultations, including the determination of priority topics, participation in the guideline development group, or external review of draft guidelines. The applicability domain required the most improvement. Facilitators and barriers during guideline implementation should be considered, which could be identified through stakeholder feedback or pilot testing before publication. The influences of this information on guideline development and recommendation formation should also be described. In addition, some tools could be used to facilitate guideline application, including summary documents, quick reference guides, educational tools, and patient leaflets. Australia 2021 (Douglas et al., 2021) used the GuideLine Implementability Appraisal (GLIA) tool to enable broader dissemination. In the Netherlands, the Antimicrobial Stewardship program is responsible for implementing and monitoring guidelines (Kullberg et al., 2017).

In summary, the evaluation items of the AGREE II instrument can assist in the development of guidelines and ensure their integrity, rigor, and applicability. Guidelines should be developed and reported with reference to all domains of this instrument to ensure higher-quality guidelines.

### 4.2 Variations on drug therapy for IA treatment and prevention

We compared the recommendations of guidelines on IA treatment and prevention published in the past decade, and found that the recommendations mainly changed with the introduction of new drugs and the update of clinical trial evidence. There is little controversy between the recommendations of different guidelines.

#### 4.2.1 Primary therapy of IA

For the primary treatment of IA, most guidelines recommend monotherapy. Combination therapy is mainly used for special cases such as severe infections, and the recommended level for initial treatment is not high.

Amphotericin B was the cornerstone of the IA treatment before 2002. In 2002, with the publication of the Global Comparative Aspergillus Study (GCAS), voriconazole gradually replaced amphotericin B in IA treatment. Until the launch of isavuconazole and the publication of the SECURE trial in 2015, the subsequent guidelines recommendations were quickly revised, making isavuconazole, along with voriconazole, the first choice for IA treatment. The GCAS study (Denning et al., 2002) was an open multicenter study that compared voriconazole with amphotericin B as the primary treatment for IA in 116 patients. Successful outcomes and survival rates at 12 weeks were higher in the voriconazole group, and voriconazole-treated patients experienced significantly fewer severe drug-related adverse events. The SECURE trial (Maertens et al., 2016) was a randomized controlled trial (RCT) that compared the primary treatment of invasive mold disease between isavuconazole and voriconazole in 527 patients. The 42-day all-cause mortality of the patients in the isavuconazole group showed non-inferiority compared with those in the voriconazole group, and the incidence of isavuconazole-related adverse events was lower.

In 2007, posaconazole showed significant overall success rate in 107 patients with invasive aspergillosis who were refractory to or intolerant of previous antifungal therapy in a multicenter study (Walsh et al., 2007). Posaconazole is therefore licensed for salvage treatment of invasive mold disease. In 2021, a multicenter RCT compared posaconazole with voriconazole as a first-line therapy of IA in 575 ITT participants (Maertens et al., 2021) and demonstrated that posaconazole was non-inferior to voriconazole for all-cause mortality and was well tolerated with fewer adverse events. This study supported the use of posaconazole as a first-line treatment for IA; therefore, the subsequent guidelines also raised the recommendation level of posaconazole for the primary treatment of IA accordingly.

L-AmB is mainly used as an alternative option when azoles are not available, especially in IA cases with primary treatment failure or azole resistance. High-dose (10 mg/kg) L-AmB demonstrated no significant benefit but was associated with higher rates of nephrotoxicity than the standard dose (3 mg/kg) (Cornely et al., 2007). Other lipid formulations of amphotericin B have no good evidence-based basis for the treatment of IA, and the recommended levels are relatively low. While the clinical status of D-AmB is gradually declining, and it is even not recommended by six guidelines due to its high incidence of adverse reactions.

There is limited data on the use of echinocandins monotherapy for primary treatment of IA, and they are mainly used as a combination therapy option. However, a RCT in 2015 evaluated the efficacy and safety of the combination of voriconazole with anidulafungin compared to voriconazole with placebo for primary IA therapy in 454patients (Marr et al., 2015). The results showed no benefit in 6-week mortality, and only *post hoc* analysis of serum GM-positive participants demonstrated lower mortality in the combination therapy arm. A network meta-analysis in 2024 (Liu et al., 2024) compared the efficacy of primary treatment regimens for IA, and the findings suggested that isavuconazole, voriconazole, and posaconazole may be the best antifungal agents for IA primary therapy, while L-AmB plus caspofungin could be an alternative option.

#### 4.2.2 Salvage therapy and breakthrough infection of IA

The current dilemma of IA treatment lies in initial treatment intolerance or failure, as well as breakthrough infections. In these cases, comprehensive analysis is needed, and some confounding factors such as immune reconstitution or co-infections need to be excluded. The inadequate concentration of azoles and azole resistance are important reasons for treatment failure. For salvage therapy, the guidelines recommend the conversion of drug types or combination therapy. Drugs with more TDM evidence include itraconazole, voriconazole, and posaconazole (Chau et al., 2021), Isavuconazole has excellent oral bioavailability and reaches predictable drug levels in adults, which may reduce the need for TDM.

Triazole resistance in Aspergillus is becoming an increasingly serious problem, especially in parts of Europe. SWAB 2017 (Kullberg et al., 2017) reported that the acquired triazole resistance of Aspergillus rapidly increased to 12.9% in 2016, with local prevalences up to 35% in specific ICU and hematology departments in the Netherlands. Azole resistance is believed to be associated with the widespread use of antifungal drugs in healthcare institutions, as well as environmental exposure to antifungal drugs in chemicals and insecticides. Azole resistance is commonly due to mutations in the cyp51A-gene that encodes the target enzyme of azoles, and the TR34/L98H and TR46/Y121F/T289A resistance mechanisms are responsible for over 80% of azole-resistant Aspergillus. The Clinical and Laboratory Standards Institute (CLSI) and the European Committee on Antimicrobial Susceptibility Testing (EUCAST) have released the breakpoints of Aspergillus. Resistance-associated gene detection in the CYP51A target enzyme or promoter have been proposed for the identification of azole-resistant *Aspergillus fumigatus* (Mellado et al., 2007). However, PCR-based assays are not currently standardized. The azole-resistant *A. fumigatus* may be resistant to multiple azole antifungal drugs or be pan-resistant. Echinocandins and L-AmB appear unaffected by the presence of an azole resistance mechanism.

The IDSA 2016 guideline (Patterson et al., 2016) does not recommend routine antifungal susceptibility testing (AFST) of isolates recovered during initial infection, while guidelines from Europe, the Netherlands, and Colombia (Kullberg et al., 2017; Ullmann et al., 2018; Oñate et al., 2022) suggested conducting susceptibility testing in patients with suspected IA as much as possible. The SWAB 2017 guideline recommends initial combination therapy with voriconazole/isavuconazole plus L-AmB, or voriconazole/isavuconazole plus an echinocandin for IA patients with unknown susceptibility to voriconazole or isavuconazole, and L-AmB Echinocandin for Proven azole resistance patients. International expert opinion on the management of infection caused by azole-resistant *A. fumigatus* in 2015 (Verweij et al., 2015) recommended that switch from voriconazole to L-AmB in confirmed IPA due to an azole-resistant Aspergillus. In regions with high rates of environmental resistance (≥10%), a voriconazole-echinocandin combination or L-AmB are favored as initial therapy.

Although combination treatment is not highly recommended in IA primary therapy by many guidelines, due to the increase of azole resistance rates, newly registered clinical studies have attempted to compare primary combination treatment with monotherapy again. The IA-DUET RCT terminated in 2024 (Lamberink et al., 2025) aims to compare azole-echinocandin combination therapy with azole monotherapy for IA. In this trial, 39 evaluable patients were included in the final analysis, the very small sample size makes a conclusive statistical analysis difficult. In addition, clinical trials of novel antifungal candidate drugs with activity for azole-resistant Aspergillus are underway, such as Olorofim and Ibrexafongerp.

#### 4.2.3 Prevention strategy of IA

The prevention strategy of IFD in high-risk patients also evolves with the emergence of clinical trial evidence. Before 2007, fluconazole prophylaxis showed a significant reduction in the incidence of invasive fungal infections in patients undergoing HSCT and became standard care (Cornely et al., 2003). In 2007, a randomized clinical trial compared the efficacy and safety of posaconazole with those of fluconazole or itraconazole as a prophylaxis for patients with prolonged neutropenia (Ullmann et al., 2007). The incidence of IA was significantly decreased and the survival rate was significantly prolonged in posaconazole group. Since then, posaconazole has been recommended as the first-line IA prophylaxis for high-risk patients. Voriconazole is also more effective than fluconazole for IA prophylaxis in HSCT recipients (Wingard et al., 2010). Among SOT patients, lung transplant recipients carry the highest risk of IA (Pappas et al., 2010), and the risk of IA in other SOT patients is much lower. Universal prophylaxis for IA is generally accepted in lung transplant recipients, and aerosolized AmB formulations have been shown to reduce the incidence of IA in lung transplant recipients (Peghin et al., 2016). Data were insufficient for routine IA prophylaxis in other SOT recipients, and prophylaxis in high-risk recipients should depend on the risk factors associated with each transplant type. Taiwan 2023 (Wu et al., 2023) with regards to CAPA recommended azoles with activity against molds for IA prophylaxis guided by risk stratification.

Despite progress in treatment, IPA still maintains a high mortality rate of over 20%, better prevention strategies may reduce the mortality loss caused by IA. An inaugural RCT in 2024 (Fortún et al., 2024) tries to examine the safety and effectiveness of nebulized L-AmB against a placebo in the auxiliary treatment of IPA. Thirteen patients with neutropenia were included, encouraging indirect efficacy data have been derived from image monitoring or biomarkers. Furthermore, a systematic scoping review of nebulized L-AmB (Hagiya et al., 2023) found that nebulized liposomal amphotericin B treatment appeared to be safe and without severe adverse effects.

### 4.3 Strengths and limitations

Our study has several strengths. First, the four assessors had extensive experience in assessing guidelines and the ICC showed high consistency, which ensured the reliability of our conclusions. Second, we used a systematic search strategy to screen IA-related guidelines published over the past decade to ensure comprehensive results. Third, this study is the first to apply the AGREE II instrument to evaluate the quality of the IA-related guidelines. Finally, we extracted and reviewed recommendations related to the drug prevention and treatment of IA.

This study also has some limitations. First, guidelines published in other formats such as books, booklets, other websites, or health institution documents might have been missed. Second, we only evaluated the methodological quality of the guidelines without specific content or original evidence. Third, we only included comprehensive guidelines for IA treatment and prevention and excluded single content-related guidelines. Guidelines for IFD prevention in hematologic malignancies were also excluded due to the lack of clear differentiation between IA and other IFD.

## 5 Conclusion

The quality of IA-related guidelines differed according to era and region. To improve their quality, future guidelines should refer to the AGREE II instrument. With the emergence of new drugs and evidence-based trials, the recommendations in the guidelines have undergone corresponding changes. Currently, voriconazole and isavuconazole are the recommended first-line therapies for IA treatment. Oral posaconazole remains the first choice for IA prevention in patients with hematological malignancies. Additional evidence-based data are needed regarding IA prevention and treatment in both chronically and critically ill patients.

## Data Availability

The original contributions presented in the study are included in the article/Supplementary Material, further inquiries can be directed to the corresponding authors.

## References

[B1] Al-Abdely H. M. Alothman A. F. Al Salman J. Al-Musawi T. Almaslamani M. Butt A. A. (2014). Clinical practice guidelines for the treatment of invasive Aspergillus infections in adults in the Middle East region: expert panel recommendations. J. Infect. Public Health 7 (1), 20–31. 10.1016/j.jiph.2013.08.003 24029495

[B2] Alanio A. Dellière S. Fodil S. Bretagne S. Mégarbane B. (2020). Prevalence of putative invasive pulmonary aspergillosis in critically ill patients with COVID-19. Lancet Respir. Med. 8 (6), e48–e49. 10.1016/s2213-2600(20)30237-x 32445626 PMC7239617

[B3] Brouwers M. C. Kho M. E. Browman G. P. Burgers J. S. Cluzeau F. Feder G. (2010). AGREE II: advancing guideline development, reporting and evaluation in health care. Cmaj 182 (18), E839–E842. 10.1503/cmaj.090449 20603348 PMC3001530

[B4] Chau M. M. Daveson K. Alffenaar J. C. Gwee A. Ho S. A. Marriott D. J. E. (2021). Consensus guidelines for optimising antifungal drug delivery and monitoring to avoid toxicity and improve outcomes in patients with haematological malignancy and haemopoietic stem cell transplant recipients, 2021. Intern Med. J. 51 (Suppl. 7), 37–66. 10.1111/imj.15587 34937141

[B5] Cornely O. A. Maertens J. Bresnik M. Ebrahimi R. Ullmann A. J. Bouza E. (2007). Liposomal amphotericin B as initial therapy for invasive mold infection: a randomized trial comparing a high-loading dose regimen with standard dosing (AmBiLoad trial). Clin. Infect. Dis. 44 (10), 1289–1297. 10.1086/514341 17443465

[B6] Cornely O. A. Ullmann A. J. Karthaus M. (2003). Evidence-based assessment of primary antifungal prophylaxis in patients with hematologic malignancies. Blood 101 (9), 3365–3372. 10.1182/blood-2002-05-1356 12393455

[B7] Dadwal S. S. Hohl T. M. Fisher C. E. Boeckh M. Papanicolaou G. Carpenter P. A. (2021). American society of transplantation and cellular therapy series, 2: management and prevention of aspergillosis in hematopoietic cell transplantation recipients. Transpl. Cell Ther. 27 (3), 201–211. 10.1016/j.jtct.2020.10.003 PMC9088165 33781516

[B8] Denning D. W. Ribaud P. Milpied N. Caillot D. Herbrecht R. Thiel E. (2002). Efficacy and safety of voriconazole in the treatment of acute invasive aspergillosis. Clin. Infect. Dis. 34 (5), 563–571. 10.1086/324620 11807679

[B9] Douglas A. P. Smibert O. C. Bajel A. Halliday C. L. Lavee O. McMullan B. (2021). Consensus guidelines for the diagnosis and management of invasive aspergillosis, 2021. Intern Med. J. 51 (Suppl. 7), 143–176. 10.1111/imj.15591 34937136

[B10] Fortún J. Gómez-García de la Pedrosa E. Martínez-Lorca A. Paredes P. Martín-Dávila P. Gómez-López A. (2024). A phase I/IIa prospective, randomized, open-label study on the safety and efficacy of nebulized liposomal amphotericin for invasive pulmonary aspergillosis. J. Fungi (Basel) 10 (3), 191. 10.3390/jof10030191 38535200 PMC10970827

[B11] Garcia-Vidal C. Alastruey-Izquierdo A. Aguilar-Guisado M. Carratala J. Castro C. Fernandez-Ruiz M. (2019). Executive summary of clinical practice guideline for the management of invasive diseases caused by Aspergillus: 2018 Update by the GEMICOMED-SEIMC/REIPI. Enfermedades Infecc. Y Microbiol. Clin. 37 (8), 535–541. 10.1016/j.eimc.2018.03.018 29960829

[B12] Gavaldà J. Meije Y. Fortún J. Roilides E. Saliba F. Lortholary O. (2014). Invasive fungal infections in solid organ transplant recipients. Clin. Microbiol. Infect. 20 (Suppl. 7), 27–48. 10.1111/1469-0691.12660 24810152

[B13] Gil L. Kałwak K. Piekarska A. Góra-Tybor J. Wierzbowska A. Bieniaszewska M. (2020). Antifungal management in adults and children with hematological malignancies or undergoing hematopoietic cell transplantation: recommendations of polish society of hematology and Blood transfusion, polish society of pediatric oncology and hematology, and polish adult leukemia study group, 2020. Acta Haematol. Pol. 51 (2), 60–72. 10.2478/ahp-2020-0014

[B14] Girmenia C. Raiola A. M. Piciocchi A. Algarotti A. Stanzani M. Cudillo L. (2014). Incidence and outcome of invasive fungal diseases after allogeneic stem cell transplantation: a prospective study of the Gruppo Italiano Trapianto Midollo Osseo (GITMO). Biol. Blood Marrow Transpl. 20 (6), 872–880. 10.1016/j.bbmt.2014.03.004 24631738

[B15] Global Action for Fungal Infections (2023). Available online at: https://gaffi.org (Accessed December 12, 2023).

[B16] Hagiya H. Nishimura Y. Otsuka F. (2023). Safety and usefulness of nebulized liposomal amphotericin B: systematic scoping review. Pulm. Pharmacol. Ther. 82, 102233. 10.1016/j.pupt.2023.102233 37414132

[B17] Husain S. Camargo J. F. (2019). Invasive aspergillosis in solid-organ transplant recipients: guidelines from the American society of transplantation infectious diseases community of practice. Clin. Transpl. 33 (9), e13544. 10.1111/ctr.13544 30900296

[B18] King J. Henriet S. S. V. Warris A. (2016). Aspergillosis in chronic granulomatous disease. J. Fungi (Basel) 2 (2), 15. 10.3390/jof2020015 29376932 PMC5753077

[B19] Kohno S. Tamura K. Niki Y. Izumikawa K. Oka S. Ogawa K. (2016). Executive summary of Japanese domestic guidelines for management of deep-seated mycosis 2014. Med. Mycol. J. 57 (4), 117–163. 10.3314/mmj.16-00010 27904053

[B20] Kullberg B. J. A.B. N. M. Janssen J. J. W. M. Meis J. F. G. Verweij P. E. Oude Lashof A. M. L. (2017). in SWAB guidelines for the management of invasive fungal infections. Editor Secretariat B.SWAB (Netherlands).

[B21] Kung H. C. Huang P. Y. Chen W. T. Ko B. S. Chen Y. C. Chang S. C. (2018). 2016 guidelines for the use of antifungal agents in patients with invasive fungal diseases in Taiwan. J. Microbiol. Immunol. Infect. 51 (1), 1–17. 10.1016/j.jmii.2017.07.006 28781150

[B22] Lamberink H. Huygens S. Aerts R. Lagrou K. van Leeuwen-Segarceanu E. Lodewyck T. (2025). Superiority trials in invasive aspergillosis: a harsh reality check with the IA-DUET (HOVON502) trial. Clin. Infect. Dis. 80 (2), 367–370. 10.1093/cid/ciae501 39378343 PMC11848251

[B23] Liu A. Xiong L. Wang L. Zhuang H. Gan X. Zou M. (2024). Compare the efficacy of antifungal agents as primary therapy for invasive aspergillosis: a network meta-analysis. BMC Infect. Dis. 24 (1), 581. 10.1186/s12879-024-09477-9 38867163 PMC11170913

[B24] Maertens J. A. Raad I. I. Marr K. A. Patterson T. F. Kontoyiannis D. P. Cornely O. A. (2016). Isavuconazole versus voriconazole for primary treatment of invasive mould disease caused by Aspergillus and other filamentous fungi (SECURE): a phase 3, randomised-controlled, non-inferiority trial. Lancet 387 (10020), 760–769. 10.1016/s0140-6736(15)01159-9 26684607

[B25] Maertens J. A. Rahav G. Lee D. G. Ponce-de-León A. Ramírez Sánchez I. C. Klimko N. (2021). Posaconazole versus voriconazole for primary treatment of invasive aspergillosis: a phase 3, randomised, controlled, non-inferiority trial. Lancet 397 (10273), 499–509. 10.1016/s0140-6736(21)00219-1 33549194

[B26] Marr K. A. Schlamm H. T. Herbrecht R. Rottinghaus S. T. Bow E. J. Cornely O. A. (2015). Combination antifungal therapy for invasive aspergillosis: a randomized trial. Ann. Intern Med. 162 (2), 81–89. 10.7326/m13-2508 25599346

[B27] Mellado E. Garcia-Effron G. Alcázar-Fuoli L. Melchers W. J. Verweij P. E. Cuenca-Estrella M. (2007). A new Aspergillus fumigatus resistance mechanism conferring *in vitro* cross-resistance to azole antifungals involves a combination of cyp51A alterations. Antimicrob. Agents Chemother. 51 (6), 1897–1904. 10.1128/aac.01092-06 17371828 PMC1891382

[B28] Oñate J. M. Rivas-Pinedo P. Saavedra-Trujillo C. H. Camacho-Moreno G. Enciso-Olivera L. Cuervo-Maldonado S. I. (2022). Section 2. Colombian consensus for prophylaxis, treatment and prevention of invasive aspergillosis in adult and pediatric patients. Infectio 26 (3), 297–339. 10.22354/24223794.1064

[B29] Pappas P. G. Alexander B. D. Andes D. R. Hadley S. Kauffman C. A. Freifeld A. (2010). Invasive fungal infections among organ transplant recipients: results of the Transplant-Associated Infection Surveillance Network (TRANSNET). Clin. Infect. Dis. 50 (8), 1101–1111. 10.1086/651262 20218876

[B30] Patterson T. F. Thompson G. R. 3rd Denning D. W. Fishman J. A. Hadley S. Herbrecht R. (2016). Practice guidelines for the diagnosis and management of aspergillosis: 2016 update by the infectious diseases society of America. Clin. Infect. Dis. 63 (4), e1–e60. 10.1093/cid/ciw326 27365388 PMC4967602

[B31] Peghin M. Monforte V. Martin-Gomez M. T. Ruiz-Camps I. Berastegui C. Saez B. (2016). 10 years of prophylaxis with nebulized liposomal amphotericin B and the changing epidemiology of Aspergillus spp. infection in lung transplantation. Transpl. Int. 29 (1), 51–62. 10.1111/tri.12679 26339864

[B32] Ruhnke M. Cornely O. A. Schmidt-Hieber M. Alakel N. Boell B. Buchheidt D. (2020). Treatment of invasive fungal diseases in cancer patients-revised 2019 recommendations of the infectious diseases working party (AGIHO) of the German society of hematology and oncology (DGHO). Mycoses 63 (7), 653–682. 10.1111/myc.13082 32236989

[B33] Shi B. Y. (2016). Chinese clinical guidelines for the diagnosis and treatment of invasive fungal diseases in 484 solid organ transplant recipients (2016 edition). Chin. J. Organ Transplant. 485, 37. 10.3760/cma.j.issn.0254-1785.2016.05.09

[B34] Singh N. Husain S. AST Infectious Diseases Community of Practice (2013). Aspergillosis in solid organ transplantation. Am. J. Transpl. 13 (Suppl. 4), 228–241. 10.1111/ajt.12115 23465016

[B35] Tissot F. Agrawal S. Pagano L. Petrikkos G. Groll A. H. Skiada A. (2017). ECIL-6 guidelines for the treatment of invasive candidiasis, aspergillosis and mucormycosis in leukemia and hematopoietic stem cell transplant patients. Haematologica 102 (3), 433–444. 10.3324/haematol.2016.152900 28011902 PMC5394968

[B36] Ullmann A. J. Aguado J. M. Arikan-Akdagli S. Denning D. W. Groll A. H. Lagrou K. (2018). Diagnosis and management of Aspergillus diseases: executive summary of the 2017 ESCMID-ECMM-ERS guideline. Clin. Microbiol. Infect. 24, E1–E38. 10.1016/j.cmi.2018.01.002 29544767

[B37] Ullmann A. J. Lipton J. H. Vesole D. H. Chandrasekar P. Langston A. Tarantolo S. R. (2007). Posaconazole or fluconazole for prophylaxis in severe graft-versus-host disease. N. Engl. J. Med. 356 (4), 335–347. 10.1056/NEJMoa061098 17251530

[B38] Verweij P. E. Ananda-Rajah M. Andes D. Arendrup M. C. Brüggemann R. J. Chowdhary A. (2015). International expert opinion on the management of infection caused by azole-resistant Aspergillus fumigatus. Drug Resist Updat 21-22, 30–40. 10.1016/j.drup.2015.08.001 26282594

[B39] Verweij P. E. Brüggemann R. J. M. Azoulay E. Bassetti M. Blot S. Buil J. B. (2021). Taskforce report on the diagnosis and clinical management of COVID-19 associated pulmonary aspergillosis. Intensive Care Med. 47 (8), 819–834. 10.1007/s00134-021-06449-4 34160631 PMC8220883

[B40] Walsh T. J. Raad I. Patterson T. F. Chandrasekar P. Donowitz G. R. Graybill R. (2007). Treatment of invasive aspergillosis with posaconazole in patients who are refractory to or intolerant of conventional therapy: an externally controlled trial. Clin. Infect. Dis. 44 (1), 2–12. 10.1086/508774 17143808

[B41] Wingard J. R. Carter S. L. Walsh T. J. Kurtzberg J. Small T. N. Baden L. R. (2010). Randomized, double-blind trial of fluconazole versus voriconazole for prevention of invasive fungal infection after allogeneic hematopoietic cell transplantation. Blood 116 (24), 5111–5118. 10.1182/blood-2010-02-268151 20826719 PMC3012532

[B42] Wu H. Y. Chang P. H. Huang Y. S. Tsai C. S. Chen K. Y. Lin I. F. (2023). Recommendations and guidelines for the diagnosis and management of Coronavirus Disease-19 (COVID-19) associated bacterial and fungal infections in Taiwan. J. Microbiol. Immunol. Infect. 56 (2), 207–235. 10.1016/j.jmii.2022.12.003 36586743 PMC9767873

