# Supplementary material for: Appraisal of guidelines and variations in recommendations on drug therapy for invasive aspergillosis prevention and treatment
Source: Front Pharmacol. 2025 Mar 31;16:1443487. doi: 10.3389/fphar.2025.1443487 (PMC11994652; doi:10.3389/fphar.2025.1443487)
Supplement: Supplementary file 1 [file DataSheet1.docx]

**Search strategy in PubMed：**

#1： ((((((((((("Invasive Fungal Infections"[MeSH Terms]) ) OR ("Invasive Fungal Infections")) OR ("Fungal Infection, Invasive")) OR ("Infection, Invasive Fungal")) OR ("Invasive Fungal Infection")) OR ("Disseminated Fungal Infection")) OR ("Disseminated Fungal Infections")) OR ("Fungal Infection, Disseminated")) OR ("Infection, Disseminated Fungal")) OR ("Invasive Mycoses")) OR ("Mycoses, Invasive") Filters: from 2013/1/1 - 2023/9/12

#2：((((("Invasive Pulmonary Aspergillosis"[MeSH Terms]) OR ("Invasive Pulmonary Aspergillosis")) OR ("Aspergillosis, Invasive Pulmonary")) OR ("Pulmonary Aspergillosis, Invasive")) OR ("Pulmonary Aspergillosis - Invasive")) OR ("Pulmonary Aspergillosis Invasive") Filters: from 2013/1/1 - 2023/9/12

#3：#1 or #2

#4：((((((Guideline [Publication Type]) OR (“Practice Guideline” [Publication Type])) OR ("Guidelines as Topic"[MeSH Terms])) OR ("Guidelines as Topic"[Title/Abstract])) OR (guideline*[Title/Abstract])) OR (guidance*[Title/Abstract])) OR (recommendation*[Title/Abstract]) Filters: from 2013/1/1 - 2023/9/12

#5：#3 and #4

**Supplementary Table A1 Grading system of evidence and recommendation.**

| **Guideline** | **Grading System Used** | **Description of Evidence** | **Description of Recommendation** |
| --- | --- | --- | --- |
| Taiwan, 2023 | GRADE | High (A); Moderate (B); Low (C); Very low (D) | Strong (1); Weak (2) |
| Columbia, 2022 | modified GRADE | High (I); Moderate (II); Low (III) | Strong; Weak |
| Australia, 2021 | GRADE | I; II; III | A(strongly); B(moderately); C(marginally); D(against) |
| Global, 2021 | GRADE | High; Moderate; Low; Very low | Strong; Weak |
| ASTCT, 2021 | Self-defined | I; II; Ⅲ | A (always be offered); B (generally be offered); C (optional); D (generally not be offered); E (never be offered) |
| Poland, 2020 | modified ECIL-8 grading system | I; II; III | A (strongly); B (moderately); C (marginally); D(against) |
| AGIHO/ DGHO, 2019 | GRADE | I; II; IIr;Ⅱt;Ⅱh; Ⅱu; Ⅱa; III | A (strongly); B (moderately); C (marginally); D (against) |
| AST‐IDCOP, 2019 | Modified GRADE | High; Moderate; Low; Very low | Strong; Weak |
| GEMICOMED-SEIMC, 2018 | GRADE | I; II; III | A (strongly); B (moderately); C (marginally) |
| ESCMID-ECMM-ERS, 2017 | GRADE | I; II; IIr;Ⅱt;Ⅱh; Ⅱu; Ⅱa; III | A (strongly); B (moderately); C(marginally); D (against) |
| SWAB, 2017 | Self-defined | Evidence level: A1(Systematic review); A2(RCT or prospective cohort study); B (comparative study, cohort study or patient-control study); C (non-comparative study); D (opinion of committee)  Evidence level: Level 1(A1, or≥2 A2); Level 2(1 A2, or≥2 B); Level 3(1 B or C); Level 4(expert opinion) | |
| ECIL-6, 2017 | ECIL grading system | I; II; III | A (good evidence to support); B (moderate evidence to support); C (poor evidence to support) |
| IDSA, 2016 | GRADE | High; Moderate; Low; Very low | Strong; Weak |
| Taiwan, 2016 | GRADE | High; Moderate; Low; Very low | Strong; Weak |
| China, 2016 | GRADE | high (A); moderate (B); low (C); very low (D) | strong (1); weak (2) |
| Japan, 2014 | Canadian Task Force | I; II; III | A (strongly); B (recommended); C1 (no scientific rationale, recommended to perform); C2 (no scientific rationale, recommended not to perform); D (scientific rationale for ineffectiveness or harm, recommended not to perform) |
| Middle East, 2014 | self-defined | A (Strongly recommended, ≥1 RCT); B (Recommended, ≥1 clinical trial; cohort or case-controlled analytic studies or multiple case series); C (Recommended, case series/reports, preclinical studies and expert opinion) | |
| ESGICH, 2014 | GRADE | I; II; Ⅲ | A (strongly); B (moderately); C (marginally); D (against) |

Evidence level: I: Evidence from at least 1 properly designed randomized, controlled trial; II: Evidence from at least 1 well-designed clinical trial, without randomization, from cohort or case-controlled analytic studies (preferably from >1 centre), from multiple time series, or from dramatic results of uncontrolled experiments; III: Evidence from opinions of respected authorities, based on clinical experience, descriptive case studies; Ⅱr: Meta-analysis or systematic review of RCT;Ⅱt: Transferred evidence, ie results from different patient cohorts or similar immune status situation;Ⅱh: Comparator group historical control; Ⅱu: Uncontrolled trials; Ⅱa: Published abstract (presented at an international symposium or meeting).

**Supplementary Table A2 Treatment recommendation for IA or IPA**

| **Guideline** | **Population** | **Recommended Treatment Regimens** |
| --- | --- | --- |
| Taiwan, 2023 | CPAP | VCZ, ISZ, PCZ, L-AmB (S/L, 1C); Alternative: D-AmB, echinocandins (S/L, 1C) |
| Columbia, 2022 | - | Primary therapy: First option VCZ, or ISZ (S/H); Alternative: L-AmB or ABLC (against D-AmB) (S/M); ITZ (S/M);  Salvage therapy: mono or combination of VCZ, PCZ, L-AmB, ABLC, or an echinocandin (CPFG, ANFG, MCFG) (S/M) |
|  | HM and/or HCT | Primary therapy: First option VCZ or ISZ(S/H); Alternative: L-AmB or ABLC (not D-AmB) (S/M); echinocandin (CPFG, ANFG, MCFG) alone or in combination(S/M); PCZ(S/M) |
|  | SOTR | Primary therapy: First option VCZ (S/H); Alternative: ISZ (S/M); L-AmB (against D-AmB) (S/M); echinocandin (CPFG, ANFG, MCFG) alone or in combination(S/M); PCZ (S/M); |
|  | SOT lung | Primary therapy: PCZ or VCZ (S/M); complementary use of nebulized L-AmB (S/L) |
|  | SOT heart | Alternative for primary therapy: ITZ (W/L) |
|  | ICU, HIV, BH | Primary therapy: First option VCZ (S/H) |
|  | CPAP | First option: VCZ or ISZ (S/M); Alternative: L-AmB (S/M) |
| Australia, 2021 | haematology/ oncology | First line: VCZ (AⅠ); Second line: ISZ (AⅠ); PCZ (AⅠ); L-AmB (BⅡ); VCZ and ANFG (CⅠ); CPFG (CⅡ); MCFG (CⅡ);  Salvage therapy: VCZ (AⅡ); L-AmB (BⅡ); PCZ (CⅡ); ISZ (CⅢ); CPFG (CⅡ); VCZ and CPFG, L-AmB and CPFG (BⅡ) |
| Global, 2021 | CPAP | First line: VCZ; Alternative: ISZ or PCZ (S/L) |
| ASTCT, 2021 | HCTR | First line: VCZ (AⅡ); Alternative: ISZ (AⅡ); PCZ (AⅢ); L-AmB (AⅡ) |
| Poland, 2020 | HM or HCT | VCZ (AⅠ); L-AmB (AⅠ); ISZ (AⅠ); ABLC (BⅡ); D-AmB (DⅡ); L-AmB and VCZ (CⅡ); L-AmB and CPFG (CⅡ) |
| AGIHO/ DGHO, 2019 | HM and/or ST | First line: VCZ (AⅠ); ISZ (AⅠ); L-AmB (AⅡ); VCZ and ANFG（BⅠ）; PCZ (CⅢ); CPFG (CⅡ); MCFG (CⅡ); ITZ (CⅢ); ANFG (DⅢ); ABLC(DⅠ); D-AmB (DⅠ); ABCD (DⅠ); Second-line/Salvage therapy: L-AmB (BⅡ); CPFG (BⅡ); PCZ (BⅡ); VCZ (BⅡ); MCFG mono or combination (CⅡ); VCZ + CPFG mono or combination (CⅡ); ABLC (BⅢ) |
| AST‐IDCOP, 2019 | SOTR | Primary therapy: VCZ (S/H); Alternative: ISZ, L‐AmB, ABLC (S/M);  Salvage therapy or first‐line antifungals are contraindicated: ANFG, CPFG, MCFG(W/L); PCZ(S/L); ITZ |
| GEMICOMED-SEIMC/REIPI, 2018 | HM | Primary therapy: VCZ and ISZ (AⅠ); echinocandins and PCZ are not recommended (AⅡ); Alternative: L-AmB (AⅡ);  Salvage therapy: L-AmB (AⅡ); echinocandins and PCZ (BⅡ) |
|  | SOTR | First line: VCZ (AⅡ); L-AmB (AⅢ) |
|  | ICU | First line: VCZ (BⅡ); Alternative : ISZ (BⅡ); L-AmB (BⅡ); against nebulized AmB as adjunctive (CⅢ)  Salvage therapy: echinocandins, preferably in combination therapy(CⅢ) |
| ESCMID-ECMM-ERS, 2017 | Neutropenia | ISZ (AⅠ); VCZ (AⅠ); L-AmB (BⅡ); VCZ and ANFG (CⅠ); CPFG (CⅡ); ITZ (CⅢ); ABLC (CⅢ); MCFG (CⅢ); ABCD (DⅠ); D-AmB (DⅠ) |
|  | Allo-HCT | ISZ (AⅡt); VCZ (AⅡt);L-AmB (BⅡt); VCZ and ANFG (CⅡt); CPFG (CⅡ); ITZ (CⅡt, a); ABLC (CⅢ); MCFG (CⅢ); ABCD (DⅡt); D-AmB (DⅡt) |
|  | HIV | VCZ (AⅢ) |
|  | SOTR | SOT heart: ITZ (CⅢ); SOT any: VCZ (AⅢ) ; L-AmB (AⅡ); VCZ and CPFG (BⅡ); if VCZ contraindicated: CPFG (BⅢ) |
|  | - | Salvage therapy: Any combination (CⅢ); VCZ (AⅡ); L-AmB (BⅡ); ABLC (CⅡ); CPFG (BⅡ); MCFG (CⅡ); PCZ (BⅡ); ITZ (DⅢ); ITZ oral forms (CⅡ) |
| SWAB, 2017 | VCZ S | First line：VCZ or ISZ; Alternative: L-AmB or PCZ; against ITZ or D-AmB |
|  | unknown S | VCZ / ISZ and L-AmB, or VCZ / ISZ and an echinocandin; Second choice: L-AmB monotherapy |
|  | azole resistance | L-AmB; Second choice: echinocandin monotherapy |
|  | ICU | azole and echinocandin or azole and L-AmB; Second choice: L-AmB monotherapy |
|  | - | Salvage therapy: L-AmB; second choice: an echinocandin |
| ECIL-6, 2017 | HM or HCT | First line: VCZ (AⅠ); ISZ (AⅠ); L-AmB (BⅠ); ABLC (BⅡ)；ABCD (CⅠ); CPFG (CⅡ); ITZ (CⅢ); VCZ and ANFG (CⅠ); other combinations (CⅢ); against D-AmB (AⅠ)  Salvage therapy: L-AmB (BⅡ); ABLC (BⅡ); CPFG (BⅡ); ITZ (CⅢ); PCZ (BⅡ); VCZ (BⅡ); Combination (BⅡ) |
| IDSA, 2016 | - | Primary therapy: VCZ (S/H); Alternative: L-AmB (S/M); ISZ (S/M); other LFAB (W/L); against an echinocandin (S/M);  Salvage therapy: LFAB, MCFG, CPFG, PCZ, or ITZ (S/M) |
| Taiwan, 2016 | - | Primary therapy: VCZ (S/H); Alternative: L-AmB(S/M); D-AmB (S/M); ITZ (W/M); PCZ (W/M); echinocandin(W/M) |
| China, 2016 | SOTR | Primary therapy: VCZ, L-AmB (1C); Critically ill: VCZ and CPFG (1C) |
| Japan, 2014 | HM | Primary therapy: VCZ(AⅠ); Alternative: L-AmB (AⅡ); Alternative or salvage therapy: CPFG (BⅡ); MCFG(BⅡ); ITZ(BⅡ) |
|  | internal medicine IPA | Suspected cases: Primary therapy: VCZ (BⅠ); L-AmB (BⅠ); ITZ (BⅡ); CPFG (BⅡ); MCFG (BⅡ);  Clinically diagnosed: Primary treatment: VCZ (AⅠ); L-AmB (AⅠ); Alternative or salvage treatment: ITZ (BⅡ); CPFG (BⅡ); MCFG (BⅡ); For severe cases, MCFG and CPFG in combination with other drugs(C1Ⅲ) |
|  | SOTR | Empirical treatment: VCZ (C1Ⅲ); L-AmB (C1Ⅲ); MCFG (C1Ⅲ); CPFG (C1Ⅲ); ITZ (C1Ⅲ)  Targeted treatment: Primary therapy: VCZ(AⅡ); Alternative or salvage treatment: L-AmB (AⅡ); MCFG (BⅡ); CPFG (BⅡ); ITZ (BⅢ) |
|  | HIV | VCZ (AⅠ); L-AmB (AⅡ); CPFG (BⅡ); MCFG (BⅢ) |
| Middle East, 2014 | - | Primary therapy: VCZ (A); Alternative: LFAB (B); CPFG (B); MCFG (B); PCZ (B); ITZ (B); ANFG (C);  Salvage therapy: ITZ (B) and echinocandins (C) |
| ESGICH, 2014 | SOTR | Primary therapy: VCZ (AⅢ); L-AmB (AⅢ); combination (AⅢ): VCZ and CPFG (AⅢ); VCZ and ANFG (AⅢ);  Salvage therapy: combination (AⅢ); VCZ or L-AmB (AⅢ); ABLC (BII); PCZ (BIII); CPFG (BIII); MCFG (BIII) |
|  | SOT lung | nebulized L-AmB or ABLC, if intolerance VCZ (BIII); if tracheobronchitis: VCZ and nebulized LFAB (AIII) |

Abbreviation: VCZ: voriconazole; ISZ: isavuconazole; PCZ: posaconazole; ITZ: itraconazole; L-AmB: liposomal Amphotericin B; D-AmB: Amphotericin B deoxycholate; ABLC: Amphotericin B lipid complex; ABCD: Amphotericin B colloidal dispersion; CPFG: caspofungin; MCFG: micafungin; ANFG: anidulafungin; LFAB: Lipid formulations of amphotericin B; ICU: intensive care unit; HIV: human immunodeficiency virus; BH: biologic therapy; S: susceptibility; S: strong recommendation; W: weak recommendation; H: high-quality evidence; M: moderate-quality evidence; L: low-quality evidence.

**Supplementary Table A3 Prophylaxis recommendation for IA or IPA**

| **Guideline** | **Population** | **Recommended Prophylaxis Regimens** |
| --- | --- | --- |
| Columbia, 2022 | - | Primary, universal and/or targeted prophylaxis: azoles (PCZ, VCZ, ITZ); Alternative: the new ITZ formulation (ITZ-SUBA, capsules) (S/H); L-AmB or ABLC, D-AmB (S/H); nebulized AmB formulations (W/L); echinocandin (CPFG or MCFG) (S/H); does not consider ISZ as an alternative (S/H);  Secondary prophylaxis: VCZ PO, CPFG followed by ITZ PO, L-AmB IV followed by VCZ PO (S/M) |
|  | SOTR | PCZ PO, or VCZ PO, or nebulized L-AmB (S/H); SOT lung: PCZ, VCZ, ITZ, D-AmB (nebulized), L-AmB (nebulized), ABLC (nebulized), echinocandin (CPFG, MCFG) (S/M); SOT heart: PCZ PO, VCZ PO, ITZ PO, echinocandin (CPFG, MCFG); SOT liver: VCZ PO, L-AmB, echinocandin (CPFG, ANFG, MCFG) (S/H) |
|  | HM and/or HSCT | Primary prophylaxis: PCZ PO or VCZ PO (S/H); HM: PCZ PO, VCZ PO, ITZ, L-AmB (nebulized + FCZ PO), echinocandin (CPFG, MCFG) (S/H); allo-HSCTR: PCZ PO, VCZ PO, L-AmB (nebulized + FCZ PO), echinocandin (CPFG, MCFG) (S/H) |
| ASTCT, 2020 | HCTR | PCZ or VCZ (AⅠ); Alternative: echinocandins (BⅠ); GVHD: PCZ (AⅠ); VCZ as an alternative to PCZ in allo-HCT (BⅠ); ISZ as an alternative to PCZ or VCZ (CⅢ) |
| Poland, 2020 | HM or HCTR | AML with intensive chemotherapy: PCZ (AⅠ); Higher-risk HCTR: VCZ (BⅠ); GVHD: PCZ (AI) |
| AST‐IDCOP, 2019 | SOT liver | ANFG, MCFG, CPFG, or VCZ (S/H); LFAB (W/M); |
|  | SOT lung | Targeted prophylaxis: nebulized D-AmB, L-AmB or nebulized ABLC (W/L); Prophylaxis or preemptive therapy: VCZ, ITZ or PCZ; Alternatives to VCZ: PCZ or ISZ (W/L) |
|  | SOT heart | Targeted prophylaxis: ITZ or VCZ or echinocandins (S/L) |
| GEMICOMED-SEIMC/REIPI, 2018 | HM and HSCT | PCZ (AⅠ); VCZ (AⅠ); ITZ (BⅡ); MCFG (BⅢ); CPFG (CⅢ); nebulized L-AmB (BⅠ); L-AmB (CⅡ) |
|  | SOT kidney | Prophylaxis is not recommended (CⅢ) |
|  | SOT liver | If criteria meeted: MCFG (AⅠ); ANFG (AⅠ); CPFG (AⅡ); L-AmB (BⅡ); ABLC (BⅡ) |
|  | SOT pancreas | All recipients: FCZ (AⅡ); If criteria meeted: MCFG (AⅡ); CPFG (AⅡ); ANFG (AⅡ); L-AmB (AⅢ) |
|  | SOT heart | If criteria meeted: ITZ (AⅡ); CPFG (AⅡ); VCZ (BⅢ); PCZ (CⅢ) |
|  | SOT lung, lung-heart | All recipients: nebulized L-AmB; nebulized ABLC (BⅡ); VCZ (CⅡ); Targeted prophylaxis: Nebulized L-AmB (BⅡ); VCZ (BⅡ) |
|  | SOT small bowel | All recipients: FCZ (AⅡ); If criteria meeted: L-AmB (AⅡ); CPFG (AⅡ); MCFG (AⅡ); ANFG (AⅡ); ABLC (AⅢ) |
| ESCMID-ECMM-ERS, 2017 | HM | PCZ (AⅠ); L-AmB nebulized with FCZ (BⅠ); ABLC (CⅡh); MCFG (C Ⅱt); L-AmB (CⅡu); VCZ (CⅡt); ITZ (DⅡ) |
|  | ALL, RIC | L-AmB（DⅠ） |
|  | Auto-HSCT or  HM besides AL | Any mould active agent (DⅢ) |
|  | Allo-HSCT (until NR) | PCZ (BⅡt); L-AmB nebulized with FCZ (BⅡt); VCZ (CⅠ); MCFG (CⅠ); ITZ (DⅠ) |
|  | Allo-HSCT (after NR and no GVHD) | Any antifungal agent (DⅢ) |
|  | Allo-HSCT (with GVHD) | PCZ (AⅠ); VCZ (CⅡ); ITZ (CⅡ); MCFG (CⅢ) |
|  | Heart SOT | Universal prophylaxis: ITZ or nebulized AmB (CⅠ); Targeted prophylaxis: echinocandins (AⅡt) |
|  | Liver SOT | Targeted prophylaxis: L-AmB (BⅢ); echinocandins (AⅠ) |
| SWAB, 2017 | neutropenia | PCZ |
| IDSA, 2016 | neutropenia | PCZ (S/H); VCZ (S/M); and/or MCFG (W/L); CPFG (W/L); ITZ (S/M) |
|  | Allo-HSCTR with GVHD | PCZ (S/H); VCZ (S/M); ITZ (S/H) |
|  | Lung SOT | VCZ or ITZ or nebulized AmB for 3 to 4 months (S/M) |
| Japan, 2014 | HM and HSCTR | ITZ (AⅡ); VCZ (AⅠ); MCFG (AⅠ) |
|  | SORT | Localized: L-AmB nebulized (BⅡ); Generalized: VCZ (BⅡ); L-AmB (BⅡ); MCFG (BⅡ); CPFG (BⅡ); ITZ (C1Ⅲ) |
| Middle East, 2014 | HM and HSCTR | First line: PCZ (A); Alternative for allo-HSCTR: VCZ (A); Pre-engraftment phase following HSCT: MCFG (A) |

Abbreviation: GVHD: graft versus host disease; AML: Acute myeloid leukemia; ALL: Acute lymphoblastic leukaemia; RIC: remission induction chemotherapy.
